# A meta-analysis and systematic review of randomized controlled trials with degarelix versus gonadotropin-releasing hormone agonists for advanced prostate cancer

**DOI:** 10.1097/MD.0000000000003845

**Published:** 2016-07-08

**Authors:** Alessandro Sciarra, Andrea Fasulo, Antonio Ciardi, Elisa Petrangeli, Alessandro Gentilucci, Martina Maggi, Michele Innocenzi, Federico Pierella, Vincenzo Gentile, Stefano Salciccia, Susanna Cattarino

**Affiliations:** a Department of Urological Sciences, University Sapienza, Rome; b Statistics Institute, Rome; c Department of Radiological Sciences, Oncology & Pathology, University Sapienza, Rome; d Department of Experimental Medicine, University Sapienza, Rome.

**Keywords:** degarelix, hormone therapy, metastatic stage, prostate neoplasm

## Abstract

Our aim was to systematically evaluate the benefits of degarelix as antagonist versus agonists of gonadotropin-releasing hormones (GnRH) for the treatment of advanced prostate cancer (PC). This comparison was performed either in terms of biochemical or oncological or safety profiles. To this end we, carried out a systematic review and meta-analysis of the literature.

We selected only studies directly and prospectively analyzing the two treatments in the same population (randomized phase III studies). We followed the Preferred Reporting Items for Systematic Reviews and meta-analyses process for reporting studies.

After we eliminated studies according to the exclusion criteria, 9 publications were considered relevant to this review. These articles described 5 clinical trials that were eligible for inclusion. The follow-up duration in all trials did not exceed 364 days. This meta-analysis and review comprised a total of 1719 men, 1061 randomized to degarelix versus 658 to GnRH agonists treatment for advanced PC. Oncological results were evaluated only in 1 trial (CS21:408 cases) and they were not the primary endpoints of the study. Treatment emerging adverse events were reported in 61.4% and 58.8% of patients in the degarelix and GnRH agonists group, respectively (odds ratio, OR = 1.17; 95% confidence interval, 95% CI: 0.78–1.77, *P* > 0.1). Treatment related severe cardiovascular side effects were reported (trial CS21-30-35) in 1.6% and 3.6% of patients in the degarelix and GnRH agonists group, respectively (OR = 0.55, 95% CI: 0.26–1.14, *P* > 0.1).

Our analysis evidences relevant limitations in particular for the comparative evaluation of the efficacy and the oncological results related to degarelix.

## 1 Introduction

Androgen deprivation therapy (ADT) is the basis of the medical treatment for advanced prostate cancer (PC) and is increasingly used in combination with radiotherapy in patients with earlier stages of disease.^[1]^ For several years gonadotropin-releasing hormones (GnRHs) agonists have been the standard of care for ADT. More recently GnRH antagonists represent an alternative form of ADT and degarelix is the main compound used and analyzed in clinical trials. GnRH antagonists demonstrated a direct and immediate action that allows castration without an initial testosterone surge or subsequent microsurges as reported with agonists.^[2]^ A comparative analysis on GnRH antagonists versus agonists on PC cases has been produced in some prospective and randomized clinical trials with more data on biochemical modifications than on oncologic results.^[3]^

Despite the relevant role of ADT in PC, most patients showing an initial response will experience progression to a castrate resistant PC (CRPC). A better choice of the compound to be used for castration and a better pharmacological sequentiality in the hormone-sensitive phase of the disease may help to delay the development of CRPC. International guidelines^[1,4]^ recommend the use of both GnRH agonists and antagonists as possible alternatives for ADT in PC. The present meta-analysis and review evaluates the comparative efficacy and safety of degarelix as specific antagonist and GnRH agonists for PC.

## 2 Methods

### 2.1 Objective

Our aim was to systematically evaluate the benefits of degarelix as antagonist versus agonists of GnRH for the treatment of advanced PC. This comparison was performed either in terms of biochemical or oncological or safety profiles. To this end, we carried out a systematic review and meta-analysis of the literature.

### 2.2 Search strategy

For each database examined, the search terms used were (“prostate neoplasm”) AND (“degarelix” OR “gonadotropin-releasing hormone antagonist”) AND (“gonadotropin-releasing hormone agonist” OR “luteinizing-releasing hormone agonist” OR “luteinizing-releasing hormone analogue”). A critical review of Embase, Medline (OvidSP), Web of Science, Scopus, PubMed, Cinahl, clinicaltrial.gov, and the Cochrane library was performed. The search was updated to July 30, 2015.

### 2.3 Inclusion and exclusion criteria

Inclusion criteria focused on men of all age groups with histologically proved PC treated with degarelix (as GNRH antagonist) versus GnRH agonists inside clinical trials. We selected only studies directly and prospectively analyzing the two treatments in the same population (only randomized phase III studies) to compare them in the most objective manner. We included and reviewed original articles, clinical trials, and reviews. In addition abstracts from trials were used only to update current information on trials already and entirely presented. There were no restrictions on the basis of years and language, but we included only trials conducted in humans. We excluded unpublished data or published only as abstracts because the information that is needed to correctly assess the study quality and results was not completely available. We also excluded reports with GnRH antagonists other than degarelix.

### 2.4 Data collection and data extraction

**Figure 1:**
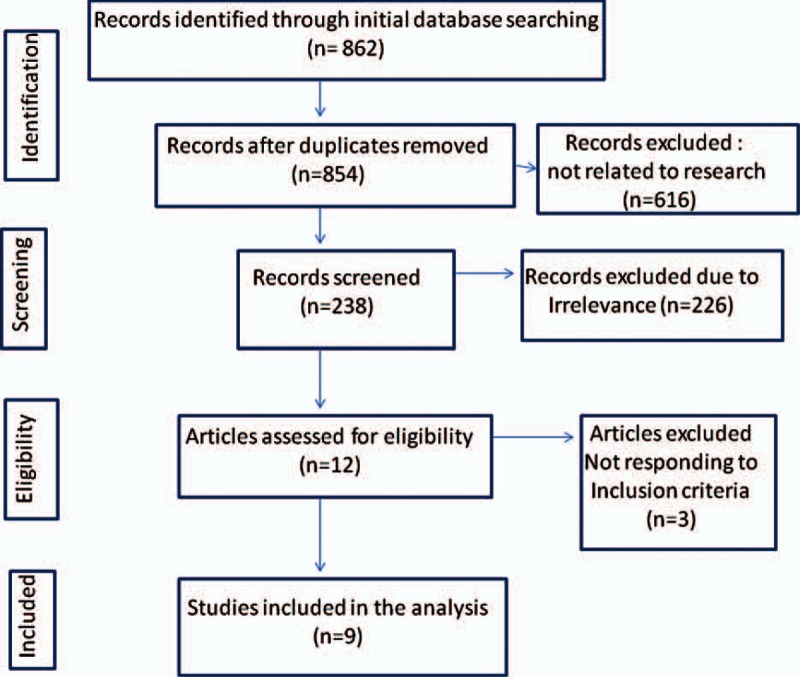
flow-chart showing the numbers of papers identified and included or excluded at each stage of our analysis.

We followed the Preferred REPORTING Items for Systematic Reviews and meta-analyses (PRISMA) process^[5,6]^ for reporting studies, with the recommended flow-chart showing the numbers of papers identified and included or excluded at each stage (Fig. 1). The entire publications were reviewed for relevance to the defined review question. The references cited in all full-text articles were also assessed for additional relevant articles.^[6]^ The search was carried out by two reviewers (AS and SS) independently. From each study, data regarding methodology, patient population, trials design, treatments, baseline parameters, biochemical assessments, oncologic efficacy assessments, safety, and quality of life analysis were extracted. We considered three populations: general population considered in the study, only nonmetastatic and only metastatic cases (when possible in the study). Data were extracted in 2X2 contingency tables.

### 2.5 Assessment of publication bias and study quality

To analyze the presence of publication bias, log-transformed values of the relative sensitivity were plotted against the associated standard errors for graphical inspection (funnel plot). In addition, asymmetry of the funnel plot was tested using the Egger regression test. Identified reports were reviewed according either to the Quality Assessment of Diagnostic Accuracy Studies (QUADAS) criteria^[6,7]^ or the Cochrane collaboration's tool.

### 2.6 Data synthesis and analysis

In each study, we compared degarelix versus GnRH agonist's outcomes either in the whole population considered or only in metastatic or nonmetastatic cases (when possible). The comparison was conducted on different profiles: biochemical (outcomes on PSA, testosterone, and gonadotropins), oncological (overall survival, PC specific survival, clinical progression frees survival, and biochemical progression-free survival), safety profile, quality of life (symptoms and quality of life questionnaires).

To synthesize the results, we performed both Fixed-Effects and Random-Effects meta-analysis comparing the two treatment regimens. A fixed-effect analysis provided a results that may be viewed as a “typical intervention effect” from the studies included. In order to calculate a confidence interval for a fixed-effect analysis the assumption is made that the true effect of intervention is the same value in each study. This assumption implies that there is no statistical heterogeneity. In case of heterogeneity a random-effect model is incorporate and it involves the assumption that the effects being estimated in the different studies are not identical. In case for all studies included also standard deviations are always available, the standardized mean difference method is used.

Survival rate was used as a binary variable in all the included studies. Therefore, the log of the odds ratio (OR) and the 95% confidence interval (CI) were reckoned as the effect size for each considered endpoint. The OR and 95% CI results could be divided into the following: (i) OR >1 and 95% noncontaining 1, rate was significantly higher in the degarelix group; (ii) OR <1 and 95% CI not containing 1, rate was significantly higher in the GnRH agonist group; (iii) OR = 1, no difference in the rates of the two groups; and (iv) 95% CI containing 1, statistically insignificant difference in the rates of the two groups. All statistical analysis were performed using R 3.2.0 (R Foundation for Statistical Computing, Vienna, Austria, 2016 (https://www.R-project.org).

### 2.7 Heterogeneity

Heterogeneity was assessed using an *X*^2^ statistic and the *I*^2^ statistic. A continuity correction was applied where necessary. If heterogeneity was not present (*P* > 0.10 and *I*^2^ < 50%), the fixed-effects model would be selected for further analysis. We performed subanalysis for two defined subgroup categories: clinically defined metastatic PC and clinically defined nonmetastatic PC.

From the studies, different GnRH agonists were used; however, we considered these treatments as a homogeneous group to be compared with degarelix treatment. We analyzed as different groups cases treated with antiandrogen only for the flare-up period or in association with GnRH agonists for all the period [complete androgen blockade (CAB)].

## 3 Results

After we eliminated studies according to the exclusion criteria, 9 publications were considered relevant to this review.^[8–16]^ These articles described five clinical trials that were eligible for inclusion in this review and meta-analysis. Data from trial CS35 were obtained only from clinicaltrial.gov^[17]^ and no extensive publications are available. All trials were prospective and randomized and the sample size ranged from 40 to 848 PC cases. Table 1 shows individual data on the methodology, patient population, treatments, baseline parameters, biochemical assessments, oncologic efficacy assessments, safety, and quality of life analysis. The follow-up duration was not the same in the included studies, but in all trials it did not exceed 364 days.

**Table 1:**
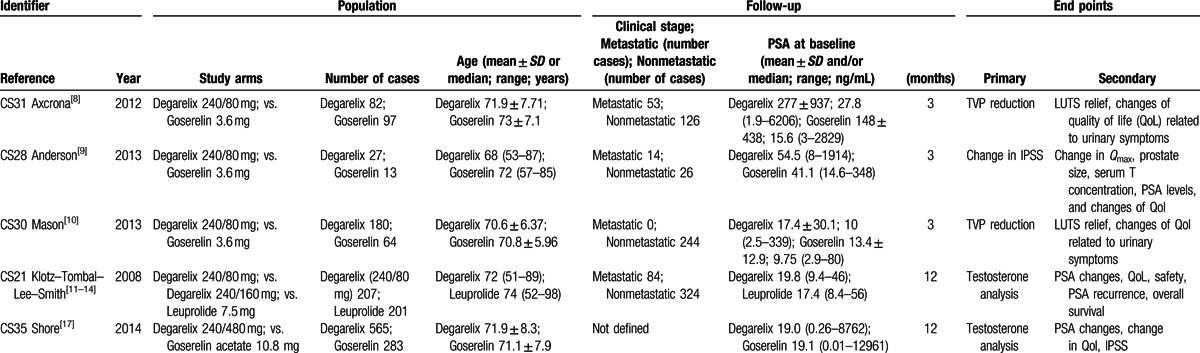
Characteristics and Populations of RCT Phase III Included in the Analysis.

Data from an extension of trial CS21 (CS21A)^[18,19]^ and trial CS35 (CS35A)^[20]^ have not been included in our analysis because they represent a crossover or an open label extension of the main trial and no new randomized comparative data are available.

This meta-analysis and review comprised a total of 1719 men, 1061 randomized to degarelix versus 658 to GnRH agonists treatment for advanced PC. A stratification of cases in metastatic and nonmetastatic PC was possible in four trials (151 metastatic and 720 non metastatic cases). All cases treated with GnRH agonists received antiandrogens only for the flare suppression and no cases were treated with CAB.

As GnRH agonist in 1 trials (CS21: 201 cases) was administered leuprolide 7.5 mg/month whereas in 3 trials (CS28-30-31: 174 cases) goserelin 3.6 mg/month and in 1 trial (CS35: 283 cases) goserelin 10.8 mg/3 months depot.

As GnRH antagonists, degarelix was administered in 4 trials (496 cases) with a starting dose of 240 mg for 1 month and thereafter monthly doses of 80 mg and in 1 trial (CS35: 565 cases) with 480 mg/3 months depot doses. In 1 trial (CS21:202 cases), there was also a third group of comparison in which degarelix was administered after the starting dose with a monthly dose of 160 mg.

### 3.1 Biochemical profile

The biochemical profile was evaluated in all trials (1719 cases) and in 2 trials (CS21-35), it was the primary endpoint. In particular data were evaluated regarding serum PSA, testosterone, and gonadotropin levels variation during treatments.

The follow-up duration was not the same in the included studies, but in all trials, it did not exceed 364 days.

Both treatments (GnRH agonists and degarelix) were able to maintain testosterone suppression to castration levels 0.5 ng/mL or less from day 28 to day 364. Castration testosterone levels were obtained and maintained for all the follow-up in 98% and 96% of cases treated with degarelix and GnRH agonists, respectively (*P* = 0.64).

From day 0 to day 28, treatment with degarelix produced castration testosterone levels in a higher percentage (97%) of cases when compared to GnRH agonists (45%; *P* = 0.02).

A meta-analysis on testosterone data is not possible because trials considered testosterone heterogeneously and not comparably.

**Figure 2:**
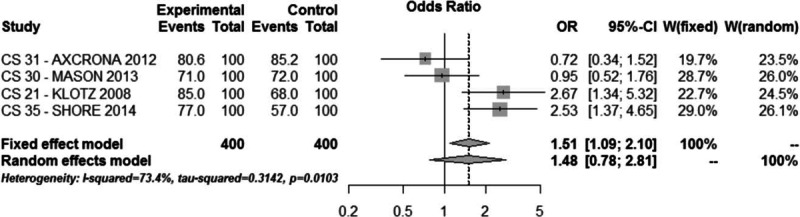
Meta-analysis of PSA variation comparing degarelix versus GnRH agonist therapy groups.

The analysis of PSA variation was possible in 4 out of the 5 trials (CS31-30-21-35). From day 0 to day 28, PSA levels declined by 78% and 71% in the degarelix and GnRH agonist group, respectively (*P* = 0.59). The differences in PSA reduction from baseline at day 28 between degarelix and GnRH agonists were not statistically significant (OR = 1.48, 95% CI: 0.78–2.81, *P* > 0.1, Fig. 2).

Only the CS 21 trial measured the differences in the reduction in FSH from baseline to the last follow-up (364 days) which were significantly higher in the degarelix (88.5% reduction) than in the GnRH agonist (54.8% reduction) group (*P* values are not reported).

#### 3.1.1 Summary of findings

Both treatments were able to maintain testosterone to castration levels to day 364. In the first 28 days, degarelix produced castration levels in a higher percentage of cases. No significant differences were found regarding PSA level variation.

### 3.2 Oncological results

Oncological results were evaluated only in 1 trial (CS21:408 cases) and they were not the primary endpoints of the study. Since just 1 trial collected oncological data, it was not possible to perform meta-analysis. Oncological results are summarized in Table 2.

**Table 2:**
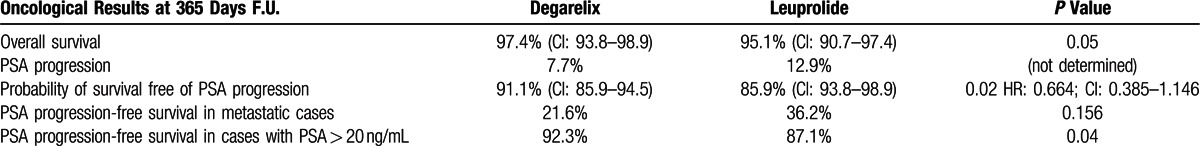
Oncological Results from Trial CS21.

In particular, data were evaluated regarding overall survival and PSA progression free survival. On the contrary, the trial (CS21) did not analyze data regarding PC specific survival and clinical progression free survival. The follow-up duration was 364 days.

Regarding overall survival, the outcomes of CS21 trial suggested that at 364 days, it was significantly (*P* = 0.05; log-rank)) higher in patients receiving degarelix (97.4%; 95% CI: 93.8–98.9) compared to GnRH agonists (95.1%; 95% CI: 90.7–97.4). The study shows that the overall causes of deaths occurred more frequently in patients receiving GnRH agonists (9 cases = 4%) compared to degarelix (5 cases = 2%). However at a limited follow-up of 364 days, the numbers of events (all causes deaths and PC related deaths) were very low in both groups and this aspect strongly reduces the significance of overall survival evaluation.

PSA progression-free survival was analyzed in a follow-up of 12 months. The outcomes from CS21 trial suggested that PSA progression occurred more frequently in cases receiving GnRH agonist (12.9%) compared to degarelix (7.7%). The probability of arriving at the final follow-up (12 months) without PSA progression was higher in patients receiving degarelix (91.1%; 95%CI 85.9–94.5) compared to GnRH agonist (85.9%; 95% CI: 93.8–98.9; *P* = 0.05; log-rank). Adjusting for baseline disease stage and PSA, this data resulted in HR of 0.664 (95% CI: 0.385–1.146).

PSA progression occurred more frequently in patients with metastatic disease in both treatment groups (21.6% with degarelix and 36.2% with leuprolide: *P* = 0.156). Moreover, PSA progression occurred more frequently in patients with baseline PSA more than 20 ng/mL in both treatment groups (7.7% with degarelix and 12.9% with leuprolide: *P* = 0.04).

The proportion of patients achieving a PSA suppression less than 4 ng/mL at day 28 was 59% versus 34% in the degarelix and leuprolide groups, respectively (*P* < 0.0001). At day 364, corresponding proportions were 83% and 78% (*P* = 0.339). Overall the proportion of patients achieving PSA less than 4 ng/mL over time was similar in both treatment groups, although achievement of PSA less than 4 ng/mL was faster with degarelix. For patients with metastatic disease, a higher proportion of those receiving degarelix achieved PSA less than 4 ng/mL over the duration of the study (percentages are not specified in the CS21 trial).

#### 3.2.1 Summary of findings

Results were mainly presented in terms of PSA progression, with a significantly higher PSA progression free survival in the degarelix group compared to GnRH agonist. PSA progression occurred more frequently in metastatic and PSA more than 20 ng/mL cases in both treatments.

### 3.3 Safety profile

**Figure 3:**
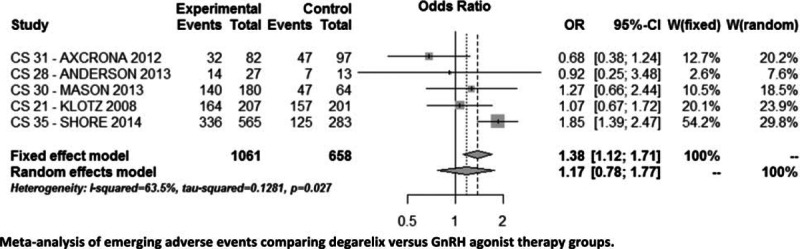
Meta-analysis of emerging adverse events comparing degarelix versus GnRH agonist therapy groups.

Safety profile was evaluated in all 5 Trials (1719 cases), but in no trial, it was the primary endpoint. The follow-up duration was not the same in the included studies, but in all trials, it did not exceed 364 days. Treatment emerging adverse events were reported in 61.4% and 58.8% of patients in the degarelix and GnRH agonists group, respectively (OR = 1.17, 95% CI: 0.78–1.77, *P* > 0.1, Fig. 3).

Most reported adverse events were mild to moderate (serious in 3% and 3.5% of cases treated with degarelix and GnRH agonists, respectively).

**Figure 4:**
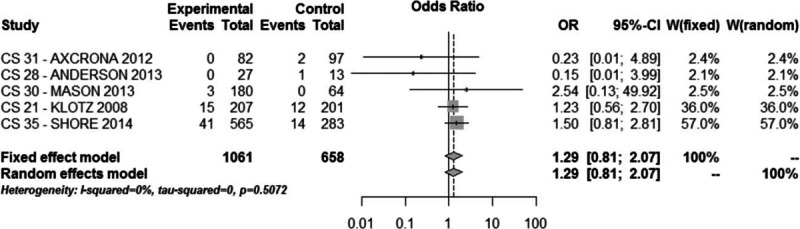
Meta-analysis of dropout from the study due to adverse events comparing degarelix versus GnRH agonists therapy groups.

Drop-out from the study due to adverse events were low in both groups (5.5% with degarelix and 4.4% with GnRH agonists; OR = 1.29, 95% CI: 0.81–2.07, *P* > 0.1, Fig. 4).

**Figure 5:**
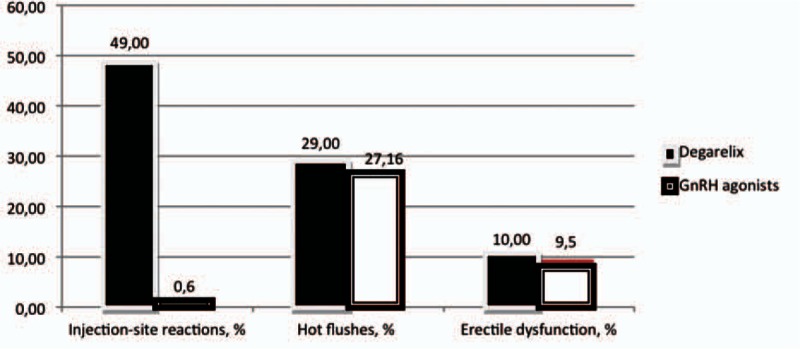
differences in the incidence of the most common treatment related adverse events.

**Figure 6:**
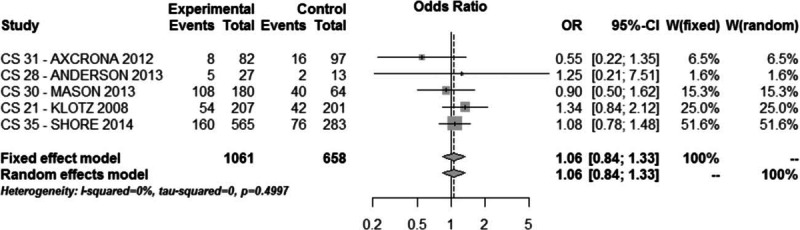
Meta-analysis of adverse event flushing comparing degarelix versus GnRH agonist therapy groups.

Differences in the incidence of each of the most common treatment-related adverse events are reported in Fig. 5. The most frequently reported adverse event was flushing (29% with degarelix and 27% with GnRH agonists; OR = 1.06, 95% CI: 0.84–1.33, *P* > 0.1 Fig. 6).

**Figure 7:**
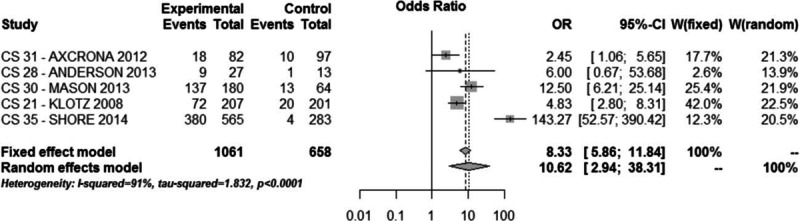
Meta-analysis of injection-site reaction adverse event comparing degarelix versus GnRH agonist therapy groups.

Degarelix was associated to a higher rate (49%) of injection-site reactions than GnRH agonists (0.6%; OR = 10.62, 95% CI: 2.94–38.31, *P* < 0.0001, Fig. 7).

**Figure 8:**
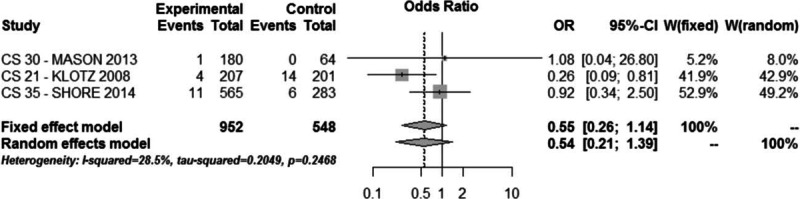
Meta-analysis of severe cardiovascular side effect comparing degarelix versus GnRH agonist therapy groups.

Treatment-related severe cardiovascular side effects (QT interval increase, angina pectoris, atrial fibrillation, cardiac failure, and myocardial ischemia) were reported (trial CS21-30-35) in 1.6% and 3.6% of patients in the degarelix and GnRH agonists group, respectively (OR = 0.55, 95% CI: 0.26–1.14, *P* > 0.1, Fig. 8).

#### 3.3.1 Summary of findings

In both groups, adverse events were mild or moderate and dropout rate was comparable and low. The main side effect related to degarelix was site injection reactions. Severe cardiovascular side effects were lower, but not significantly, in the degarelix group.

### 3.4 Symptoms and quality of life analysis

Symptoms and quality of life were analyzed in 4 trials (CS28-30-31-35: 1311 cases) and in 3 trials (CS28-30-31), they were the primary endpoints. In all trials the IPSS questionnaire was used; in one trial (CS35) also the BPH Impact Index and the SF-12 /SF-36 were reported.

The follow-up duration was not the same in the included studies, but in all trials it did not exceed 364 days.

**Figure 9:**
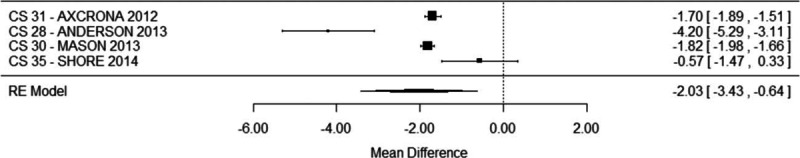
Meta-analysis of LUTS variation comparing degarelix versus GnRH agonist therapy groups.

Lower urinary tract symptoms (LUTS) estimated by the IPSS questionnaire, showed a higher decrease in the degarelix (5%) than in the GnRH agonists (3%) group during the follow-up (MD = −2.03, 95% CI: −3.43 to 0.64, *P* < 0.01, Fig. 9).

**Figure 10:**
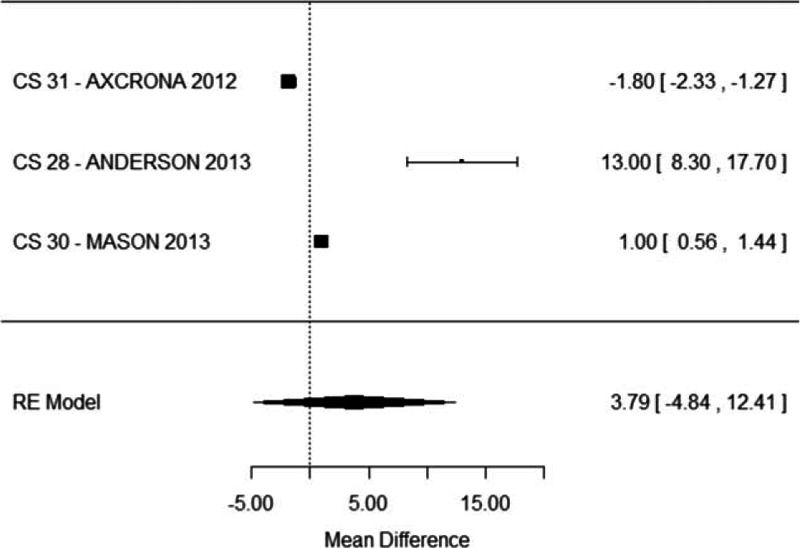
Meta-analysis of prostate volume reduction comparing degarelix versus GnRH agonist therapy groups.

The reduction of prostate volume after 90 days (last follow-up in which it was analyzed) was similar in the degarelix (38%) and in the GnRH agonist (34%) group (MD = 3.79, 95% CI: −4.84 to 12.41, *P* = 0.38, Fig. 10).

Improvement in quality of life was differently expressed in the 4 trials and a meta-analysis of data is not possible. However all studies reported a significantly (*P* < 0.05) higher improvement in the degarelix group versus the agonist group.

#### 3.4.1 Summary of findings

LUTS showed a significantly higher reduction in the degarelix group whereas prostate volume reduction was similar compared to GnRH agonist.

## 4 Discussion

For many years GnRH agonists have been the ADT standard of care. There are, however, different drawbacks related to the mechanism of action: in particular the initial testosterone surge delays the development of castration levels. Such as for GnRH agonists, several GnRH antagonists have been synthesized and analyzed. However, most of clinical trials and international guidelines recommendations are referred to degarelix.^[1–4]^ For this reason, we limited our meta-analysis to randomized clinical trials comparing degarelix to GnRH agonists.

Our aim was to systematically evaluate the benefits of degarelix for the treatment of advanced PC. This comparison was performed either in terms of biochemical or oncological or safety profiles. To this end we carried-out a systematic review and meta-analysis of the literature. We only selected studies directly and prospectively analyzing the two treatments in the same population (randomized phase III studies), to compare them in the most objective manner. However the source of the heterogeneity observed in our meta-analysis is related to different characteristics of trials: population, follow-up, and different choice of GnRH agonist.

Our review identified several articles on this topic; 9 papers were considered relevant to this review and these describe only 5 clinical phase III trials that were eligible for inclusion in our meta-analysis.^[8–17]^ On the basis of the limited number of trials and the limited population included in some of these (ranged from 40 and 848 cases), we decided to do not stratify results on the basis of the type of the agonist used (leuprorelin and goserelin). Moreover, only a comparison between degarelix and GnRH agonist associated to antiandrogen for the flare up suppression was possible. We have no randomized data comparing degarelix with a CAB.

Our analysis confirms that most of significant information from the randomized clinical trials is referred to the biochemical profile of the two treatments and this was the primary end point in 2 trials. All trials showed that both treatments (GnRH agonists and degarelix) were able to maintain testosterone suppression to castration levels to day 364. Unfortunately, the long-term maintenance of testosterone castration was randomly analyzed only for the first 12 months of treatment. From day 0 to day 28, using degarelix, castration testosterone levels are reached rapidly with no surge. Improved short-term testosterone control with degarelix can have immediate implications, in particular in terms of symptoms. Symptoms are another well-studied parameter with data from 4 trials (1311 cases) and in 3 trials they were the primary endpoints. The comparative analysis of symptoms is mainly based on the IPSS questionnaire and our meta-analysis showed a significantly higher decrease in LUTS in the degarelix than in the GnRH agonists group during the follow-up (MD = −2.03, 95% CI: −3.43 to 0.64, *P* < 0.01).

It should be relevant to have more randomized data on other categories of symptoms (related to the oncological progression), such as more structured data on the quality of life profile. Lee et al.^[13]^ analyzed quality of life improvement from trial CS21.Using the SF-12 questionnaire authors suggested that degarelix, slowing PSA progression may improve patient health related quality of life. However, when controlling for utility estimates and factors expected to be influenced by treatment (adverse events and PSA progression), there was no significant effect of treatment (degarelix versus leuprolide) on quality of life.

Some studies^[10]^ suggested a possible advantage of degarelix on GnRH agonists in the neoadjuvant use related to radiotherapy primary treatment. No data from randomized trials significantly supports this hypothesis. Our meta-analysis showed that the reduction of prostate volume (that may represent an advantage for the following radiotherapic treatment) after 90 days was similar in the degarelix and in the GnRH agonist group (MD = 3.79, 95% CI: −4.84 to 12.41, *P* = 0.38).

A second well studied biochemical profile is that related to PSA variations. This analysis was possible in 4 out of the 5 trials. In the CS21 trial as well as faster testosterone suppression, degarelix was associated with significantly faster PSA reduction versus leuprolide. However, our meta-analysis, summarizing data from all 4 trials, showed that the differences in the reduction in PSA from baseline between degarelix and GnRH agonists at day 28 were not statistically significant (OR = 1.48, 95% CI: 0.78–2.81, *P* > 0.1).

The safety profile of the two compounds is reported in all 5 trials. Unfortunately, we can obtain comparative data only during the first year of treatment and we cannot compare a long-term safety. Our meta-analysis shows a similar good profile for degarelix and GnRH agonists with a low rate of drop out due to adverse events. The only significant difference was related to injection-site reactions rate that was significantly higher with degarelix (OR = 10.62, 95% CI: 2.94–38.31, *P* < 0.0001).

Several analyses in the literature suggest a better cardiovascular safety profile for degarelix.^[14,21]^ Our meta-analysis shows that treatment related severe cardiovascular side effects (QT interval increase, angina pectoris, atrial fibrillation, cardiac failure, and myocardial ischemia) are specifically reported in 3 trials on a follow-up no longer than 12 months. The incidence of these events considered all together, was lower in degarelix (1.6%) than in GnRH agonists (3.6%) group but a statistical significance was not reached (OR = 0.55, 95% CI: 0.26–1.14, *P* > 0.1).

Smith et al.^[14]^ analyzing data only from CS21 trial, reported no significant QT modifications between the two treatments but a lower incidence of ischemic heart disease in the degarelix (4%) than in the leuprolide (10%) group. In an open noncomparative analysis,^[21]^ authors reported that cardiovascular event rates were similar (*P* > 0.1) before and after degarelix treatment in the total population and in men without cardiovascular diseases at baseline. In contrast, event rates were higher after degarelix treatment in men with cardiovascular diseases at baseline (*P* = 0.0013).

### 4.1 Limitations from the analysis of oncological results

In particular two review articles^[3,15]^ sustain the significance of oncological results from degarelix when compared to GnRH agonists. Klotz et al^[15]^ analyzing data from the 5 randomized clinical trials, reported a higher rate of PSA progression-free survival with degarelix than with GnRH agonists, either in the whole population (HR 0.71, *P* = 0.017) or in patients with baseline PSA more than 20 ng/mL (HR: 0.74; 95% CI: 0.55–1.00; *P* = 0 = 0.052). Klotz et al.^[15]^ described also a better overall survival with degarelix (HR = 0.47; *P* = 0.023) than with GnRH agonists.

Van Poppel et al.^[3]^ similarly supported the oncological advantage of degarelix versus GnRH agonists, in particular for cases with baseline PSA levels more than 20 ng/mL. They included in their analysis the CS21A open-extension, so to have a longer follow-up at a median of 27.5 months.

Our analysis evidences relevant limitations in particular for the comparative evaluation of the efficacy and the oncological results related to degarelix. The oncological evaluation is based only on one trial (CS21) and it was not the primary endpoint. Data from trial CS35 are not extensively published and oncological results are not clearly available from clinical trial.gov.^[17]^ It is not possible to include in a comparative randomized evaluation the CS21A extension.^[18,19]^ In this trial the randomized period remains related to the first year of treatment (CS21 trial)^[12]^ and after there is a not useful crossover and all cases are treated with degarelix.

The survival analysis performed by trial CS21 tended to support an advantage of degarelix on GnRH agonist either in terms of PSA progression free survival or overall survival. However, many and relevant uncertainties exist. The population included is mixed, considering either nonmetastatic or metastatic cases and this aspect can reduce the statistical power of the study. The most limiting aspect is the follow-up of the trial (only 365 days). It is not possible to extend this follow-up using the CS21A trial: after the first year no comparative randomized data are available. During this follow-up, the numbers of events and in particular the numbers of deaths are limited, so that overall survival cannot be significantly evaluated.

Therefore, no meta-analysis can be performed regarding oncological results and, regarding overall survival, the limited follow-up (only 12 months) and the limited number of events do not consent to evaluate advantages from one treatment to the other.

Always related to the limited follow-up, a PSA progression was mainly described only in metastatic and baseline PSA more than 20 ng/mL cases. In these cases, the probability of arriving at the final follow-up without PSA progression was higher in patients receiving degarelix (91.1%; 95% CI: 85.9–94.5) compared to GnRH agonist (85.9%; 95% CI: 93.8–98.9; *P* = 0.05; log-rank). Adjusting for baseline disease stage and PSA, these data resulted in HR of 0.664 (95% CI: 0.385–1.146).

In the main CS35 trial, participants were randomized 2:1 to treatment with degarelix or goserelin, respectively. All participants who completed the CS35 trial after initiation of the CS35A were eligible to enroll into this extension, provided that their treatment could continue uninterrupted. Patients entering the CS35A trial continued with the same 3-monthly treatment as they received in CS35 (i.e., degarelix 480 mg or goserelin 10.8 mg). It was intended that patients in CS35A would receive treatment with degarelix or goserelin for a period of 40 months (including 13 months’ treatment in CS35). It was, however, decided to prematurely terminate the CS35A trial due to an insufficient number of patients being enrolled. When the trial was closed 156 cases in degarelix and 80 cases in goserelin group were present. Maximum exposure of treatment was 111 weeks (in both treatment arms).^[20]^

The primary end-point of CS35A was PSA progression free survival differences at 3 years of treatment. Degarelix was to be considered noninferior to goserelin if the upper limit of the two-sided 95% CI of the adjusted hazard ratio was less than or equal to the noninferiority margin of 1.33. The results of the trial showed an HR = 0.774 (95% CI = 0.542–1.106; *P* = 0.1589) that was consistent with a noninferiority of degarelix on goserelin. Stratification of data on the basis of tumor stage or baseline PSA is not available.^[20]^

The trial reported also the HR for mortality rate at 3 years between degarelix and goserelin, but the hypothesis of noninferiority was considered null (HR = 0.595; 95% CI = 0.259–1.368; *P* = 0.2212).^[20]^

## 5 Conclusions

Our meta-analysis showed the following results: (i) both treatments were able to maintain testosterone to castration levels to day 364. In the first 28 days, degarelix produced castration levels in a higher percentage of cases. No significant differences were found regarding PSA level variation. (ii) In both groups, adverse events were mild or moderate and drop-out rate was comparable and low. The main side effect related to degarelix was site injection reactions. Severe cardiovascular side effects were lower, but not significantly, in the degarelix group. (iii) LUTS showed a significantly higher reduction in the degarelix group whereas prostate volume reduction was similar compared to GnRH agonist. (iv) The evaluation of the oncological efficacy of degarelix in randomized trials is strongly limited by the 12-month follow-up. Results were mainly presented in terms of PSA progression, with a significantly higher PSA progression-free survival in the degarelix group compared to GnRH agonist. PSA progression occurred more frequently in metastatic and PSA more than 20 ng/mL cases in both treatments. In particular for high-risk or metastatic cases, the advantage or the noninferiority of a treatment (degarelix on GnRH agonists) should be demonstrated in terms of overall survival or clinical progression-free survival as primary endpoints.
