# A meta-analysis and systematic review of randomized controlled trials with degarelix versus gonadotropin-releasing hormone agonists for advanced prostate cancer: Erratum

**DOI:** 10.1097/MD.0000000000005671

**Published:** 2016-12-09

In the article “A meta-analysis and systematic review of randomized controlled trials with degarelix versus gonadotropin-releasing hormone agonists for advanced prostate cancer”,^[1]^ which appeared in Volume 95, Issue 27 of *Medicine*, the fourth line of Table 2, “PSA progression-free survival in metastatic cases” was given incorrectly. The entry should have read “PSA progression rates in metastatic cases”.
